# On the Convulsive Affections of Infants and Children

**Published:** 1848-02

**Authors:** Marshall Hall

**Affiliations:** Foreign Associate of the Royal Academy of Medicine of Paris, etc., etc.


### SELECTIONS

FROM

### AMERICAN AND FOREIGN JOURNALS.

*On the Convulsive Affections of Infants and Children.* By Marshall Hall, M. D., F. R. S., Foreign Associate of the Royal Academy of Medicine of Paris, etc., etc. (Read before the Medical Society of London, May 17th, 1846.—I need not inform the members of this venerable society that I have been for many years engaged in an investigation of the nature and physiological application of a certain principle of action and series of phenomena in the nervous system, which I have designated excito-motor and reflex, and limited to the spinal centre, with its associated incident and reflex nerves, exclusively of the cerebral and ganglionic systems.

But it *is* necessary to state that the subject of this investigation has *practical* application to the diagnosis, pathology, and treatment of a certain class of diseases in which a tithe is not yet known.

It is a specimen of this application of physiological science to practice, to which I now beg to call the attention of this Society, the members of which will, I am persuaded, receive in good part the observations and suggestions which I am about to lay before them. It is lamentable to observe the general supineness of the profession in regard to improvements which require a little unbiassed attention—a profession, the objects and aims of which are so elevated and noble, but of which the welcome of new scientific truths must be admitted to be tardy, and the approbation niggard. In medicine alone, improvement is without recompense.

But science ought to be pursued for its own sake, irrespective of consequences, and the reward should be in the conscience. It is with these views that I beg, this evening, to submit to this Society the following observations on the nature of the convulsive affections of infants and children,—observations which are the first—or amongst the first—of a series of contributions from *the Science of Physiology to the Art and Practice of Medicine.*

It has long been the habit of the indolent in our profession to decry physiology, and especially experiment. I will venture to affirm, that such persons are incapable of grasping the question I am about to discuss—viz: the nature, diagnosis, and treatment of convulsive diseases and their effects. The greater part of this question is involved in the recent investigation relative to the excited reflex actions, and he who has not witnessed, or whose eye is not familiar with, these phenomena, as displayed in experiments, is utterly unprepared to comprehend, and to treat adequately, the class of convulsive diseases. Books cannot convey this knowledge. Books do not contain it. We have only to read the attempts to apply the principle of the excited reflex actions to practice, even by those who might seem to know something of the matter, to perceive the utter insufficiency of book-knowledge for this purpose.

No. He who would conscientiously perform his duty as a physician, in these affections, must submit to the drudgery, often fraught with pain, as well as toil, of reading the book of Nature. He must have both his eye and his mind tutored to distinguish between the effects of volition, and the phenomena of emotion, and the reflex actions of the excito-motor power.

With an eye so tutored, there is no danger of mistaking the phenomena of convulsive diseases. All is plain, simple, useful. The practical physiologist, the physiological physician, sees, that strabismus, the contracted finger, the contracted toe, are one and the same kind of affection, in different parts of the muscular system; that the partial closure of the larynx, inducing laryngismus stridulus, and the total closure of the larynx, suspending the respiration; that strangury and tenesmus, are of the same nature, only seated in other and more vital or important organs; lastly, that the general convulsions of the face, and of the general frame, are still similar morbid phenomena, differing only in their degree of diffusion and intensity, and in being of centric origin. He perceives that it would be as unphilosophical and unreasonable to think and speak and treat of the condition of the larynx separately, and to view the case as one of partial paralysis, or of inflammatory action, as so to view the affection of the muscles of the eyeball, the fingers, or the toes, which differ in no respect from that spasmodic affection of the larynx, except in their less formidable import.

To the eye of the practical physiologist, and to no other, the *series* of symptoms in convulsive disease presents fearful *excited*, *reflex* actions in the human subject. *He* sees how peculiar and specific they are in their character, and how peculiarly and specifically they are connected with the true spinal system, anatomically and physiologically. In a word, *he* alone possesses the secret cipher by which this mysterious page of Nature may be read. We might as well pretend to understand fever itself from books, as physiology, with its practical applications. Such is the nature of practical physiology; such the inconsiderate folly of those who, in the present day, as in the day of the immortal Harvey, decry wise, judicious, well-devised experiments.

The time is coming—though it may yet be distant—when the "mere practical man" will be viewed as the mere empiric, which he is, in fact; and when to know the nature and modes of action of the springs of life will be accounted the appropriate preparation for the investigation, prevention, and treatment of diseases.

If I wished to set forth, in the most vivid manner, the great reality and importance of the recent progress in our knowledge of the nervous system, and its influence on pathology and practice, I should appeal to the condition of medical opinion on the subject of the convulsive diseases of infants and children twenty years ago and at the present time. Cerebrum was confounded with spinal marrow; the sympathetic explained everything, and yet, as Professor Muller has sarcastically observed, nothing. Even recently, it has been a question whether the symptom termed laryingismus, though forming a part of a general convulsive or spasmodic affection, and being of a transitory character, be of the nature of paralysis or of spasm!

§ 1. SUBJECT OF INVESTIGATION.

There is no more terrible disease than infantile convulsion —none more deplorable in its consequences to mind, or limb, or life; for the little patient may remain an idiot, or a cripple, or the subject of future epilepsy, even should life be spared. Well, then, does this theme merit the attention of this Society.

The first question which presents itself is—What are the causes or sources of infantile convulsive affection?—in what modes are such affections induced?

The second question is—What are the precise forms assumed by these convulsive affections?

The third—What are the effects of these affections on other organs?

The fourth—What is the diagnosis of the different forms of convulsive disease?

The fifth—What are the modes of prevention and treatment?

### § 2. THE TERMS EMPLOYED.

Various terms have been employed to denote the various forms and symptoms of convulsive disease; and *one* especially has been so employed as to appear to give to one form of this affection a distinct existence and importance—I mean that of *laryngismus;* and this has led to much confusion and error in treating of this affection.

It is, in fact, as if we were to treat of cough, or any other mere symptom of disease, as a distinct disease.

Laryngismus is a symptom of disease, often a very severe one, but still only a symptom. It has been further distinguished by the epithet *stridulus* which, however, is unnecessary, if we be only well informed on the subject. There is only one kind of laryngismus, and this one is not always stridulous. In its worst and most dangerous form, indeed, it is a *silent*, because a complete, closure of the larynx.

The term laryngismus may be retained. Without intention on his part, the proposer of it chose a designation which associates itself very appropriately with that of another partial form of convulsive disease—viz: strabismus. And other symptoms of convulsive affection may receive designations which will assimilate equally well together and with these; such are chirismus and podismus, for the spasm of the wrists, the hands, or the fingers, or of the feet or toes, or the carpopedal spasm, as these have been called; whilst the term sphincterismus may be used to designate the spasmodic affections of the sphincter ani, or neck of the bladder, assuming the form of tenesmus or strangury.

Let this termination in *ismus* be used only to designate a *symptom*, and that of a purely nervous or spasmodic or convulsive character.

Laryng*ismus* will henceforth be viewed merely as a symptom, as cough is a symptom, to be traced to its origin,—to the special disease on which it depends,—and will no longer be confounded with laryng*itis*, as I fear it still is by some practitioners.

A reform in nomenclature is at once the usual result of progress in medical science, and the efficient cause of improvements in the art and practice of medicine.

§ 3. THE PREDISPOSITION.

The predisposition to convulsive affection, and especially to that form of it which has been designated laryngismus, is sometimes so marked, that all the children of a numerous family have been successively affected with this disease. Such an instance was published, by a father, in the Lancet, in April, 1841. Such instances have been observed by all physicians of experience.

Whether this predisposition be hereditary, or be induced by external circumstances, has not, perhaps, yet been accurately determined. I suspect that the effects of *locality*, as a damp soil, has frequently constituted the predisposing cause of this affection, and that a total change of residence is as important as a corrective of this predisposition as a change of air has appeared to be in the actual attacks; and this both for the safety of the individual patient and that of future children of the same parents.

But I must also add that I have known the children of different branches of the same family to be liable to this affection, though their localities have been distant from each other.

§ 4. THE CAUSES.

No irritation of the substance of the cerebrum or cerebellum can produce a muscular spasm or contraction *immediately*. This fact is the result of all experiments on animals, and and of all observations on the human subject. No disease, therefore, *limited*, in itself and in its effects, absolutely to the cerebrum or cerebellum, can be the cause of convulsion.

But irritation of the *membranes* within the cranium produces various muscular contractions—a fact which I ascertained in an important experiment, which I carefully detailed in the second series of my "Observations and Suggestions in Medicine," p. 64. No doubt *disease* sometimes acts as an irritant to these membranes, and produces its peculiar effects or symptoms—a fact for which we are indebted, with the light which it throws upon diagnosis, to experimental physiology.

6 *

But it is when a source of irritation is applied to the medulla oblongata, or medulla spinalis, that the most energetic or frightful forms of muscular contractions, or of convulsions are induced.

Lastly, when certain nerves connected with the spinal marrow, which I have designated the incident spinal nerves, are irritated, especially at the points of their origin in the cutaneous, mucous, or other tissues, various muscular contractions, now designated excited and reflex, occur. Excited through the medium of these incident nerves, the spinal centre sends forth its influence along certain muscular nerves especially associated with the former, and peculiar muscular actions ensue.

Infancy is the most excitable period of human existence, whilst it is exposed to its own peculiar sources of irritation.

Of these, one is the peculiar condition of the gums and of the alveolar processes which occurs during dentition. It is one of great vascular action and fulness, and of consequent tumefaction and pressure on the dental nerves.

Another excitement frequent in the age of infancy is that arising from the condition of the stomach, in cases in which food has been given in quantity or quality not easy of digestion. The gastric acid is then secreted in undue measure, adding this source of irritation to that of indigestible aliment.

It is a singular fact, that the age of convulsive disease is specially that of dentition, and of what I may term dubious diet. Dentition, as I understand the term, begins at birth, or even before; it is then that the tissues of the alveolar processes and cavities become the seat of augmented vascular action and nervous excitement, which do not cease until dentition is completed. I think it probable that many convulsive affections and their dire effects have their origin *in utero;* certainly many of the latter are congenital.

A third source of irritation is that which arises from morbid matters retained in the lower part of the alimentary canal, especially undue acidity, undigested food, flatus, scybala.

It is a well-known fact, that there is a greater mortality amongst children deprived of their nurse and natural food, and, as the phrase is, 'brought up by the hand,' than amongst children that are duly nursed. Whether this greater mortality depends on the greater prevalence of *convulsive* diseases is not so well determined. This is a question still requiring careful investigation.

It is difficult to determine the best kinds of food for infants deprived of the breast. Diluted cow's milk, but especially asses' milk, would seem the most natural, whilst the wholesomeness of *farinaceous* food is at least questionable.

A fourth source of excitement is that of the atmospheric air itself, and especially of the north, east, or north-east winds; and perhaps certain vapors, or the exhalations in cer tain localities.

The influence of certain states of the external atmosphere is probably exerted on the incident nerves of the larynx itself, which are thus excited, so as to induce, through reflex action, partial or complete closure of that organ. How important, then, is it to protect the susceptible little patient from an influence so fraught with danger!

With this influence of inspired air may be compared that of water, or other fluids, given to drink.

It is especially in this manner that the cold, dry air of the north-east wind, or the cold, damp air of certain localities, inspired, or cold fluids taken into the pharynx, may act in inducing laryingismus, especially in the cases of undue susceptibility, whether hereditary or induced, which obtains in this malady.

It is precisely what is observed in *choking* from any cause. I know a patient in whom the larynx and pharynx are so excitable, that, for a long time, he has been unable to swallow cold water, or use even warm water, as a gargle without laryngismus. A drop of water, or a particle of bread, falling on the rima glottidis, produce a precisely similar, but more severe, effect in all, i. e., laryngismus or even laryngismus stridulus.

The north or east wind, a cold and damp atmosphere, certain vapors in the atmoshere, etc., act on the unduly susceptble incident laryngeal branches of the pneumogastric nerve, and induce its *closure* by reflex action; the dashing of cold water on the face acts on the tri-facial nerve, with which are mysteriously associated in action, the muscles which *open* that organ. How perilous, under such circumstances, the attempt to give the little infant *anything* to swallow.

Mr. Barlow observes—"I have seen four cases in which drinking brought on the paroxysm. Hot or cold fluid was more excitant than lukewarm. In one instance, the last attack was induced in this manner; in previous attacks, there had been crowing; in this, the glottis seems to have been instantly closed, and convulsions and death speedily followed. In another, I observed that the attack was induced by sleep, excitement, pleasure, vexation, or the act of swallowing."

I have watched the effects of these causes, singly and combined, too carefully to doubt that they may, severally or together, produce or renew convulsive affections in infants. It is difficult to determine the specific action of each, but certainly, whilst strabismus, or the spasmodic contraction of the hand or foot, may arise from teething, and from gastric or intestinal irritation, the condition of the larynx is very apt to be affected by the north-east winds, or other conditions of the atmosphere, and the sphincters, by the contents of the rectum and perhaps of the bladder.

A little experience will teach us, in a case so involved in obscurity and doubt as to the individual cause, most carefully to avoid *all;* this conclusion is of the greatest moment in practice.

I think it highly probable, fifthly, that the occurrence of this convulsive affection may become associated with an augmented excitability of the spinal centre; and that it is from this cause, that the attacks are apt to be reproduced by causes too slight to produce such an effect originally. Hence, the modes of treatment found to be beneficial, should be continued carefully, *long* after the little patient *appears* to be well. This caution I also regard to be of great practical importance.

### § 5. THE INFLUENCE OF SLEEP.

The condition of the nervous system, during sleep, is peculiar. It is not unallied to epilepsy, and hence, it often passes into epilepsy, and especially into convulsive affection, in infants. The influence of volition being withdrawn, or nearly so, the muscles of the neck and larynx, like the orbicularis, contract on the venous system distributed in the neck, and the breathing becomes audible; it also ceases to be rhythmic; and during its first and deeper stage, attacks of laryngismus, or other forms of convulsive disease, in infants, as of asthma or epilepsy in adults, are apt to occur.

In deep sleep, there is a low degree of laryngismus even, manifested by stertor or snoring.

During sleep, the nervous centres are congested. If we open the eye, the conjunctiva is often observed to be deeply suffused.

In this condition of the circulation, we need not feel surprised if undue excitability occur, and that a dream, or an excitant of reflex action, may add to the peculiar condition of the larynx and of respiration, which I have described, that which constitutes actual laryngismus.

§ 6. CEREBRAL DISEASE.

I have already stated, that no disease of the cerebrum, limited in its effects to this organ, can induce convulsive affection; for no irritation of the cerebral substance can produce muscular contraction.

It is only when, by its locality, it can irritate the membranes, the incident or motor nerves, or the medulla oblongata; or when, by its magnitude, it can produce compression of that medulla, that it can produce such effect.

Inflammation, or tubercular granulation or tumor, with serous effusion at the base of the brain, may act in the former manner; and effusion into the ventricles, or inflammation, or congestion, in the substance of the cerebrum, may act in the latter.

One source of congestion is the violent fits of cough in pertussis; another is equally the cause and result of general convulsion.

In all these cases the cause of convulsion resides within the cranium, and our attention must be directed to subdue morbid action in this cavity.

§ 7. EXCITED REFLEX ACTIONS.

In by far the greater number of cases the symptoms of convulsive affection are reflex actions. As stammering would scarcely exist without emotion, so laryngismus, for example, is most effectually avoided by avoiding *every* exciting cause of reflex action.

These causes are, as I have already stated, principally *four*:

1. The irritation of the *trifacial* nerve in the gums and alveolar processes in dentition.

2. The irritation of the pneumogastric nerve by indigestible matters in the stomach.

3. The irritation of spinal nerves from morbid contents of the intestines.

4. The influence of the external atmosphere, under certain circumstances, on the incident branches of the pneumogastric nerve of the larynx, etc.

The influence of all these causes of reflex action is more or less *continuous;* and hence arises a peculiarity in their effects, which are more or less persistent. The strabismus, though not absolutely constant, frequently continues for a considerable period; and the chirismus and podismus are apt to assume a tetanoid character.

Some of the symptoms are, indeed, transient in their duration, as the laryngismus. This difference is explained on the principle of interference; the acts of respiration interfere with the tendency to closure of the larynx; at this age volition only slightly interferes with the closure of the fingers or toes; the laryngismus is therefore less permanent, than the chirismus or podismus; but when the laryngismus *is* continuous, it induces a fatal asphyxia.

The convulsive affection would also seem to wear out the excitability of the spinal system—as is seen distinctly in certain experiments—and so to cease, although the exciting cause may not be removed, and no principal of interference exists.

Such being the character of these reflex actions, it is extremely interesting to observe what are the precise organs affected. These are precisely those which are bound, as it were, by the chain of special incident and reflex nerves with the spinal centre, and constitute the spinal system. The larynx, the sphincters, are special examples of this fact; as are, I believe, certain symptoms connected with the gall-ducts. The morbid actions of the muscles of the eyeballs, of the fingers, of the toes, and in the general convulsion of the countenance, and of the general frame, obviously point out the special part of the nervous system first and specially affected, and the mode and character of the affection.

I appeal to my fellow-members of this Society, whether we were prepared to pursue the investigation of this momentous subject, before the recent views of the spinal system and its associated incident and reflex nerves with their special centre, and with their excito-motor and reflex modes of action, were laid before the profession. Whereas now, a case of convulsive disease, whatever its seat and form, is as intelligible to us as the simplest and best devised experiment, possessing the additional and almost tragic interest of an experiment replete with a thousand dangers on a human being, the object of the keenest affections, hopes and fears!

I must now add that this malady has sometimes appeared to affect other branches of the pneumogastric nerve besides the laryngeal—viz: the bronchial, and the branches distributed to the minutest branches of the air-cells, the gastric, the hepatic, and the nephritic, inducing peculiar effects. These effects have not hitherto been duly traced. But I beg to call the attention of the members of this Society to the interesting topic. Bronchitic, gastric, hepatic, and venal symptoms frequently form a part of this convulsive affection; the flow of bile is arrested; the urine is frequently affected with a deposit of the lithates. Are these cause or effect, or independent of the other phenomena?

Through this labyrinth, the knowledge we now possess of the spinal system can alone guide us. Like the stethoscope, it enables us to apply another, and in this instance, a new and internal sense, to the investigation and diagnosis of disease.

§ 8. OF LARYNGISMUS SPECIALLY.

Without closure of the larynx—extreme laryngismus, and the consequent congestion of the nervous centres — there could, I believe, be no convulsion! This closure of the larynx must be complete, in the affection under consideration, as in all others, before convulsion *can* take place. Hence I have known the actual convulsion long warded off, by constantly and carefully watching the little patient, and whenever laryngismus was threatened, dashing cold water on the face. In this manner, too, the late Dr. Denman prevented the accession of puerperal convulsions in a case of the deepest interest, of which the details are given in his valuable work.

In pertussis, there is no convulsion, however violent the fits of coughing, until extreme laryngismus or closure of the larynx occurs.

In bronchitis, and in asthma, there is no convulson, because there is no laryngismus.

Laryngismus is the efficient cause of convulsion in epilepsy. It is the first link in the fearful chain of symptoms in this fearful malady. To Mr. Barlow I am indebted for the details of a case of epilepsy, in which the extreme laryngismus and closure of the larynx was preceded by stridulous laryngismus, precisely similar to the crowing inspiration of infants.

It is laryngismus which destroys the patient in hydrophobia.

It is laryngismus which destroys the patient in the case of poisoning with strychnine.

The extent of the fearful influence of laryngismus in disease has not been appreciated in or by the profession. It is laryngismus which proves the cause of sudden death—which has occasionally induced dismay in the practitioner, and unjust reflections on the part of the patient's friends—in cynanche tonsillaris, and in scarlatina anginosa.

It was by merely witnessing the phenomena of strangulation in hydrophobia, that the late Dr. Physick of Philadelphia, was induced to suggest the operation of tracheotomy in this dire malady—a suggestion full of promise, if, indeed, there be hope at all in this disease.*

Juster views of the pathology of epilepsy will at least prevent us from attempting to give anything, far less any irritant or excitant, as cold water, or sal volatile, in the fit of epilepsy. In epilepsy, as in the crowing inspiration of infants, extreme laryngismus is the cause of sudden death. When this does occur, it may be induced by the attempt to give even cold water. Mr. Barlow was recently informed of such a fatal event, arising from this very cause, acting in this very manner, in the case of a little patient on his list, at the Infirmary for Children.

I believe a tube worn in the trachea would render the attack of epilepsy impossible! Extraordinary idea.

### § 9. EMOTION, PASSION.

Many facts lead to the conclusion that emotion and passion *act*, not through the cerebral system of nerves, but the spinal and ganglionic systems. Hence these affections of the infantile mind are the frequent cause of various convulsive affections, and especially of strabismus and laryngismus.

Hence, too, the importance of procuring a judicious, careful, and patient nurse, of excellent temper herself, and able to maintain a state of mental tranquillity in her little charge.

I have frequently observed a momentary strabismus when an infant is brought into the room, which ceased soon afterwards.

Under the same or similar circumstances, a momentary laryngismus also not unfrequently occurs.

External noises, disturbance of any kind, fretfulness and passion, however induced, are the frequent cause of strabismus, or of laryngismus—this last sometimes, in its se-

*See the Life of P. S. Physick, M.D., by J. Randolph, M.D., &c., 1839, page 58.

verest form is attended by asphyxia, and not free from danger.

The fear of this is the true and real objection to the use of the gum-lancet; otherwise there is *none*. The question must be resolved, in each individual case, by carefully watching its effects. I have often thought, that as a thermometer might be employed to determine the condition of the dental circulation, a lancet might be concealed, in a ring, or so as to have none of the formidable appearance of the ordinary gum-lancet.

But the objection which applies to lancing and scarification of the gums applies with equal force to the administration of medicine, enemata, and almost every kind of remedy.

There is ample scope in such cases for the judgment of the physician. The infantile mind must be cared for as well as the body; and whilst every cause of reflex action is carefully removed, every source of mental fret and irritation should be as carefully avoided.

It is at the moment of awaking, especially, that these precautions are most required. It is on awaking that every source of surprise, every noise, every cause of excitement, should be specially avoided; it is at this moment of repaired excitability that spasmodic or convulsive movements are most apt to be excited.

The important and extensive influence of emotion in exciting disease or its paroxysms, is still untraced. Amongst mental causes, it is emotion which induces insanity; it is not, for instance, the calculations, but the hopes and fears, of the speculator, which derage his reasoning faculty, and set imagination at fault. The paralysis agitans is induced originally, and throughout its course, by emotion. What an interesting topic for investigation.

### § 10. CONVULSION, ASPHYXIA.

In the actual convulsion the larynx is closed with a forcible effort of expiration, the respiration being suspended; and there are violent actions of the muscular system—of the eyes, face, and general frame.

These muscular actions are rarely equal on the two sides of the mesial plane, and therefore the eyes, countenance, and general frame, are greatly distorted, and the aspect of the little patient is frightful.

The venous system of the head and face is greatly distended, (in epilepsy in the adult, I have known this condition issue in ecchymosed spots.)and the countenance appears tumid and livid. The eyes, the lips, the tongue, and doubtless the venous and capillary systems of the cerebrum, the thymus, &c., partake of the same condition.

The aspect of the countenance is very different, according as the attack assumes the character of convulsion, or that of asphyxia; in the former it is livid and purple, with distention of the veins on the temple and forehead, and of the capillaries; in the latter it is livid and pallid, and cold and clammy.

These two conditions should be carefully distinguished, though they are doubtless more or less combined in every case.

The violent convulsion leaves a state of congestion of the cerebrum, with the danger of effusion, or actual effusion.

This state of asphyxia leaves the blood deteriorated.

### § 11. AUGMENTED EXCITABILITY.

I will take the liberty of detaining the Society a few minutes whilst I state a few facts on the subject of augmented excitability in the nervous centre, in a certain class of diseases.

If a mild voltaic current be made to pass through the denuded and insulated spinal marrow, or lumbar nerves, in the frog, for ten or twenty minutes, and then be withdrawn, the lower limbs are thrown into a state of tetanoid spasm. This condition of the nervous centre or muscular nerve I have designated the *electrogenic.*

If, instead of affecting the nervous tissue in this manner, we place the frog in a dilute solution of strychnine for a similar period, the spinal marrow being divided near the occiput, we have again a tetanoid condition of the limbs.

These two tetanoid conditions are not, however, the *same*, or even similar: the first is permanent, and requires for its manifestation no excitant; the second is one of predisposition only, and exists only when induced by some external exciting cause; in the former, the electrogenic state is the excitant; in the latter, the strychnogenic condition, if I may use this term, is not an actual excitant but only one of induced augmented susceptibility to other excitants.

I believe these two conditions exist in certain peculiar states of disease in the human subject, of which they are therefore, the appropriate *types;* spinal arachnitis, a spicula of bone irritating the spinal marrow, produces effects similar to the former; a lacerated cutaneous nerve, (inducing tetanus), and a poisonous state of the blood, (in hydrophobia), produce a condition similar to the latter.

All this is a deduction from experiment; I commend it to the attention of my brethren in the profession.

I now return to the subject of convulsive affections in infants.

The spinal centre becomes so excitable in some of the cases of this convulsive affection, that causes which have no influence, or no injurious influence in health, produce marked effects in the course of this malady.

I have already observed that the little patient, on being brought into the room in which the physician, a stranger, is waiting, generally or frequently manifests the effects of the slight emotion in a transient strabismus or laryngismus. The slightest fret of mind, or sudden fret of mind, or sudden start, produces a similar effect.

A mere change of room, or of locality, or of the direction of the wind, will often renew the malady, even when it has ceased for a time. On the other hand, "air and exercise" are the great antidotes to augmented excitability. Change of air, the exposure to the air when the weather is fine, are as essential as security against its inclemencies.

During the progress of dentition, it frequently happens that repeated attacks of this affection, in its varied forms, are experienced. It is, therefore, apt to be protracted through many months, indeed, until dentition is completed.

I fear that this excited condition may be protracted even through future years, and that the convulsive affection of the infant may lead to epilepsy in the adult. Fearful idea! The attacks of this very epilepsy begin with a species of laryngismus,—an actual closure of the larynx,—and proceeds, in its own manner, to produce the general convulsion, and the other formidable effects of this dire malady.

It is singular enough, and the idea that this laryngismus belongs to a peculiar nervous system, cannot be repeated too often, that, in the excitability induced by strychnine, it is a species of laryngismus which is the peculiar symptom, when its effects are severe or fatal, in the human subject, or in the warm-blooded animal. In the extreme case it is, as in the extreme laryngismus, instant asphyxia.

This laryngismus, so far from being peculiar as an affection of infants, is, as I have already stated, common to a whole class of affections of the true spinal system: emotion, hysteria, epilepsy, tetanus, hydrophobia; one and all affect the larynx especially; one and all produce a state of varied laryngismus. This affection of the larynx, therefore, instead of being a disease, or a symptom of disease, is a symptom denoting an affection of a certain nervous *system.*

The true idea of the morbid affection under consideration, as of tetanus itself, is that of an excitant or excitants, which first induce a state of augmented spinal excitability, or *erethismus;* then, various excited reflex actions; and thirdly, general convulsion.

Tetanus and hydrophobia consist in an induced spinal erethismus, the paroxysms consisting of augmented or excited spasm, from external excitants.

The infant under the influence of what I will designate the convulsive tendency, susceptibility, or erethismus, may therefore be *compared* to the patient affected by tetanus. The causes are similar,—irritated nerve, inducing spinal erethismus; and this, susceptibility to excited reflex actions; the effects are analogous—tetanoid affections.

Not dissimilar in nature is the state of things in the augmented excitability which is induced by strychnine, and which occurs in hydrophobia.

In the infantile disease, as in tetanus, the augmented susceptibility is indeed induced through irritated *nerve;* whilst in the effects of strychnine, and hydrophobia, it is induced by a poison in the *blood* itself—important distinction in the pathology of this class of diseases, first pointed out in my New Memoir, published—if, indeed, it can be said to be published—in 1844. In both sets of cases, the disease consists in an induced augmented excitability of the spinal marrow, or *spinal erethismus* of the spinal marrow, whilst its paroxysms are the result of excited reflex action, until we arrive at general convulsion of the nervous centres.

### § 12. EFFECT ON THE CEREBRUM.

Let actual convulsion once occur, and all is changed and complicated. It is no longer the spinal system only which is affected; the cerebrum has become congested, and effusion is threatened; and now double care is required to prevent the wreck of intellect or of limb, from the influence of pressure, or of counter pressure, of the organ of mind itself, or of the tissue occupying the base of the cranium—the membranes, trifacial and pneumogastric nerves, the medulla oblongata.

What a study for the physician enlightened by physiology! How extensive, how complicated! How are we to be guided through this labyrinth? By experiment. The tissues, to which I have referred, must be irritated and compressed, one by one, and the respective effects noted with the utmost care and attention; and the results must then be compared with the events witnessed in actual practice.

We may imagine what is the precise condition of the brain and of the medulla oblongata, and indeed of the interior circulation generally, by carefully observing the congested state of the veins, and of the capillary vessels of the *face*.

We should also carefully examine the condition of the *bregma*, or fontanelle: it is concave, plane, or convex, affording an index to the condition of the circulation, or it may be effusion, within the cranium.

### § 13. SUDDEN DISSOLUTION.

Sudden death may take us by surprise at *any* period of convulsive disease.

This event may be of two kinds: the first is asphyxia, from extreme laryngismus; the second is what I have designated secondary asphyxia, and probably arises from the circulation of blood imperfectly arterialized in the coronary arteries.

Extreme laryngismus may occur quite unexpectedly amongst the first symptoms, or in the midst of such general convulsive affection as may have repeatedly taken place, and passed off without much apparent danger. If but a slight strabismus, or a slight contraction of the finger, remain; or even if all symptoms have entirely disappeared, fatal laryngismus may still occur, taking us by surprise, all having appeared well and safe.

I do not think we have any means of judging *when* this event is more likely to take place than usual.

Our prognosis, therefore, however justly tinctured with hope, should always be most guarded, in this class of maladies; otherwise our judgment will assuredly be sometimes painfully called into question.

The prevalence of a northeast wind appears particularly apt to induce laryngismus in its extreme form; and in this manner several cases of sudden death have occurred in practice nearly at the same period of time.

A fit of passion is frequently the immediate cause of closure of the larynx, and of dissolution. And though I never knew so disastrous an event to occur, I have often feared lest it might take place during the use of the gum-lancet; and it is precisely the real danger of exciting a paroxysm of convulsive affection, which is the real, and I may add, the sole, objection to the use of this important remedy.

In reference to such an event, Capuron remarks—"L'enfant a été réellement suffoqué." Dr. Johnson, of Dublin, saw "a child in a state of asphyxia, caused by this disease, recovered from apparent death by the instantaneous application of artificial respiration."

In the case of threatening of asphyxia, the remedy is—*instantly* to dash cold water on the face and other parts of the general surface. The further treatment, in general, is that for *asphyxia*, especially the institution of artificial respiration. The thorax and abdomen should be speedily compressed, and then be left to the force of resilience, in order that inspiration, however slight, may follow. Frictions of the limbs, with pressure, directed upwards, should be used energetically.

The douche, the attempt at artificial respiration, the frictions, should be repeated and pursued, however apparently ineffectual, with *perseverance.*

Tracheotomy was suggested by Dr. Hugh Ley, and it was successfully performed by Dr. Johnson, in this emergency. On learning this afterwards, Dr. Ley added the following note:—

"I have made no essential alteration in my statement, that the grounds upon which I was originally disposed to advocate the propriety of the operation may remain upon record, and as a proof of the value of well-conducted physiological experiments in illustrating pathological facts, and suggesting remedial expedients. It is a reply to the maudlin sensibility, with regard to such experiments, too prevalent at the present time."—*On Laryngismus Stridulus*, p. 273.

The cause of sudden death in this class of maladies, is, however, not always mere laryngeal asphyxia. It is sometimes, as I have already stated, of that kind which may be termed secondary, or cardiac asphyxia—a condition which arises from the frequent partial closure of the larynx, and consequent partial asphyxia. The blood is then not sufficiently oxygenated or decarbonized; and as in *all* morbid conditions of the *blood*, there is still more sudden death than in the worst extreme forms of laryngismus. The poisoned blood acts on the fibres of the heart, destroying their irritability. Death is instantaneous, and I fear there is neither time, nor hope, for remedies. Arrested respirations leave a few minutes; arrested circulation scarcely a moment.

Sir Benjamin Brodie mentioned a case to me in which sudden death had occurred in a case of tetanus, after all tetanic symptoms had disappeared. The patient had been in a bath, and was dressing, saying that he was quite well! What was the rationale of this sudden event? Was it exhausted excitability? Does the same principle hold in the convulsive diseases of infants?

### § 14. THE DIAGNOSIS.

The diagnosis in the convulsive diseases of children is—

1. That of the kind or *origin* of the disease, and especially that between the *centric* and *ex-centric* affection.

2. That of the *form* of the disease, and especially that of the different, partial, and general convulsive affections; for it may be so partial as to consist of one symptom only, as strabismus laryngismus, or it may be general, and frightfully convulse every part of the muscular system.

In the *centric* affection there are generally pain, and cerebral symptoms, such as affections of the sleep, temper, and senses—wakefulness, fretfulness, intolerance of light and noise, and a peculiar contraction of the brow—from the beginning.

In the *ex-centric* affection, there is, at the first, no cerebral symptom: all the phenomena are spinal: general convulsion must take place before cerebral symptoms are observed.

The diagnosis between that part of this affection designated laryngismus and laryngitis, is founded on two circumstances:

1. The transitory character of the symptoms in the former, and its permanency in the latter; and

2. The complication of the former with strabismus, chirismus, and other convulsive or spasmodic affection.

The same principles of diagnosis distinguish spasmodic laryngismus from any paralytic influence or compression of the pneumogastric nerve on the larynx, in which case there may be other effects of paralysis of the pneumogastric nerve, especially accumulated secretion in the bronchial tubes, and pulmonary tissue, leading to cough and various '*râlés.*' The reality and the unbiassed diagnosis of this form of disease are still to be ascertained.

Laryngismus induced by bronchitis, or any inflammatory affection of the trachea or larynx, acting as an irritant on the incident laryngeal nerves, would be distinguished by the same absence of other spasmodic affection.

§ 15. POST-MORTEM APPEARANCES.

The post-mortem appearances depend entirely on the nature and course of the disease.

If the case be one of those rare instances of affection of the cerebrum or its membranes, leading to irritation or compression of the medulla oblongata, intra-cranial incident nerves, or the membranes in which certain of these are embedded, the morbid appearances will be such as are usually found in these cases. There will be *cerebral* symptoms.

In one case the medulla oblongata was distinctly indurated.

If there have been repeated violent convlsions or fits of pertussis, we shall perceive cerebral congestion or effusion, and with this probably tumefaction of the *thymus* gland.

But in those cases in which no repeated general convulsions had occurred, and in which the little patient had died suddenly perhaps, no marked appearances of the nervous centres may be detectable. The disease in this respect, as in so many others, resembles asphyxia, or tetanus.

The condition of the gums and alveolæ, of the stomach, of the intestines, of the lungs, the liver, the kidneys, should be carefully examined; but of the morbid appearances in these nothing is known.

§ 16. PREVENTION AND TREATMENT.

I now come to the last and most important topic of my paper—the prevention and treatment of convulsive diseases; to which, indeed, the views which have been given immediately lead, and in the course of which they serve as a torch to enlighten our path.

The first thing to be accomplished by the physician, as in all other cases in practice, is a full and accurate diagnosis of the disease, its form, its simplicity or complexity; its effects; and especially whether there has or has not occurred general convulsion.

If the case be one of centric origin, which is the more rare, the original disease must, of course, be treated energetically. If it be of ex-centric origin, or reflex, which is by far the more frequent case, the excitant or excitants, whatever these may be, must be carefully sought out, removed and avoided.

But *as a rule*, in *all* cases, the influence of *all* excitants, *all* excitants of emotion, of reflex action, must be absolutely removed. For even in the centric affection, it may be undue excitability only which is induced, and the *attacks* may depend upon external excitants.

The augmented arterial action within the gums and the alveolar processes, must be subdued by deep, diffused, and repeated scarification of the gums, conducted with every precaution, to avoid excitement of a mental kind.

The stomach should be emptied forthwith. This may frequently be readily done by irritating the fauces with a feather, or the finger; or a dose of ipecacuanha may be given; and then such diet should be administered, according to such rules, as may prevent the presence and delay of undigested matters in the stomach. A new and healthy nurse, or asses' milk, given by means of the bottle, are resources of the utmost moment.

The intestines should be promptly washed out by means of ample enemata of tepid water, and they should then be kept well relieved, gently free indeed, by means of mild but efficient, aperient medicine.

I have great reason to suspect the existence of undue *acidity*, not only in the stomach, but in the course of the intestinal tube, in those cases; and I strongly recommend *antacid* aperients, such as a combination of the bicarbonate of potassa, and the carbonate of magnesia, in the proportion of one-fourth and three-fourths, in some proper aromatic or aperient vehicle, and repeated so as to produce the double effect of neutralizing the gastric acid and moving the bowels.

The next object is, to guard the little patient against every injurious impression from the external atmosphere. When the northeast winds prevail, or the air is cold and damp, the patient's bed should be surrounded, at intervals of about one foot, by *three* distinct curtains or tents of gauze, or of net; the air of the room should be protected from partial currents, be well supplied with hygrometric moisture, and be maintained at a temperature of 65° Fahr.

Every mental disturbance must be avoided; the approach of a stranger, the administration of the gum lancet, and, not less, of medicine or other remedies, must be managed as carefully as possible.

The *sleep* should be *watched;* if it be disturbed by dreaming or starting, the infant should be gently awakened, and any sudden noise or light should be avoided; precautions necessary, indeed, at all times.

As stammering would scarcely exist without emotion, so the convulsive diseases of infants and children, especially those of ex-centric origin, would scarcely exist without emotion and excitants of reflex action; an aphorism of the utmost moment in practice, and admitting of great extension; for, in this respect, with the affection under consideration, chorea, the paralysis agitans, tetanus, and even hydrophobia itself, may be ranked, in some degree.

If laryngismus should exist and be extreme, the larynx being closed, water must be forcibly sprinkled on the face; the larynx is opened by the new excitant acting on other nerves and muscles, and inspiration is excited.

If apparent asphyxia have taken place, and this measure have been tried in vain, artificial respiration should be attempted: the chest and abdomen should be compressed, and the pressure should be suddenly removed, (I once witnessed asphyxia from this cause in a puppy. I applied my ear so as to examine the beat of the heart; the pressure induced expiration, and inspiration followed on its removal, and the puppy recovered. Or the lips of the practitioner should be applied to the mouth of the infant, whilst its nostrils are closed, and its trachea pressed against the œsophagus: in a word, every measure should be adopted to which we have recourse in other cases of asphyxia.

If general convulsion be threatened, or have occurred, every precaution and measure should be adopted which can protect the cerebrum from congestion and its effects: the alcoholic lotion applied to the head, leeches, cupping, mercurials, and purgative medicines, fomentations and warmth applied to the feet, &c., must all be employed with promptitude and energy.

The secretions must be attended to—the bile, the urine especially. If the former be deficient, the use of warm water, enemata, should be doubly enforced. If the urine be effected with lithate deposits the antacid aperients must be doubly enjoined.

The hydrocyanic acid, hyoscyamus, &c., may also prove useful.

§ 17. CONCLUSION.

And now, Sir, I beg to draw this paper to its conclusion. If I have, in the course of my observations, vindicated the importance and necessity of physiology—experimental physiology—to the practical physician;—if I have shown the value of the recent investigations into the nervous system, in the investigation, diagnosis, and treatment of diseases, I have fully attained the object which I had proposed to myself, in bringing this paper before you and my fellow members of this society, and I beg to thank you for your kind attention to its rather lengthy details.

I will conclude by adducing the words of one of the most logical and eloquent writers in the English language.

"In proportion as any branch of study leads to important and useful results—in proportion as it gains ground in public estimation—in proportion as it tends to overthrow prevailing errors,—in the same degree it may be expected to call forth angry declamation from those who are trying to despise what they will not" (for cannot) "learn, and wedded to prejudices which they cannot defend. Gallileo would probably have escaped persecution if his discoveries could have been disproved, and his reasonings refuted. The same spirit which formerly consigned the too powerful disputant to the dungeon or the stake is now, thank Heaven! compelled to vent itself in railing, which you need not more regard than the hiss of a serpent which has been deprived of its fangs."*

I know not what treatment could have induced the learned author to write these words; but I venture to affirm that it was not more iniquitous than that which has been inflicted on the THEORY—of *hypothesis* there is none—which has guided me throughout the discussion set forth in this paper, which may be viewed as a specimen of what may be done for the whole class of the diseases of the nervous system.

The best, the only reparation, must consist in the publication of such an Essay as the present one, in which the value and importance of the doctrine are shown in its practical

* Lectures on Political Economy by R. Whateley, D.D., Archbishop of Dublin.

application to pathology and therapeutics. That doctrine—the doctrine of the distinct spinal system; of incident and reflex nerves, in special conjunction with their spinal centre—the excitor nature; the reflex form of its actions—the special organs and functions under its control:—that doctrine—the anatomy and the physiology of the spinal system, has, in effect, become pathology and therapeutics! The nerves, the actions, the functions involved, are the same in health and in disease; an experiment has been made to present the type of a malady, and to teach us what we ought to do to succor our patient, when in extreme suffering, and in the utmost peril!

All this is depicted—placed before the eye itself—in the admirable plates which, thanks to Mr. Simpson, of Stamford, accompany my New Memoir, published, that is, printed and calumniated, (*technically*, "written down,"—see the British and Foreign Medical Review,) in 1844, which does contain something new, something which neither the preceding Memoirs, nor any other preceding book whatever, contains. Such is the power of calumny—for, in this respect, I am compelled to differ from the eloquent author whose words I have last quoted—that this Memoir is as if it were unpublished! The serpent, slander, is *not* yet "deprived of its fangs," and it is cold-blooded and long-lived, as well as venemous. I can only repeat my conviction that there are, in that volume, principles which will lead to great improvement in our practice, rejecting calumninous reviews.

I have pursued experimental physiology. I have applied the results to the mitigation of human suffering. I only wish the profession to receive my offering in good part, and to examine for themselves.

